# Perinatal mortality associated with use of uterotonics outside of Comprehensive Emergency Obstetric and Neonatal Care: a cross-sectional study

**DOI:** 10.1186/s12978-016-0241-x

**Published:** 2016-10-06

**Authors:** Louise T. Day, Daniel Hruschka, Felicity Mussell, Eva Jeffers, Stacy L. Saha, Shafiul Alam

**Affiliations:** 1 LAMB MIS-Research Department, Parbatipur, Dinajpur 5250 Bangladesh; 2 LAMB Hospital Pediatric Department, Parbatipur, Dinajpur 5250 Bangladesh; 3 Arizona State University, Tempe, AZ 85287 USA; 4 LAMB Hospital Obstetric Department, Parbatipur, Dinajpur 5250 Bangladesh

**Keywords:** Perinatal mortality, Uterotonics, Bangladesh, Stillbirth, Neonatal death, Child survival, Under-five mortality

## Abstract

**Background:**

Prior studies have shown that using uterotonics to augment or induce labor before arrival at comprehensive Emergency Obstetric and Neonatal Care (CEmONC) settings (henceforth, “outside uterotonics”) may contribute to perinatal mortality in low- and middle-income countries. We estimate its effect on perinatal mortality in rural Bangladesh.

**Methods:**

Using hospital records (23986 singleton term births, Jan 1, 2009-Dec 31, 2015) from rural Bangladesh, we use a logistic regression model to estimate the increased risk of perinatal death from uterotonics administered outside a CEmONC facility.

**Results:**

Among term births (≥37 weeks gestation), the risk of perinatal death adjusted for key confounders is significantly increased among women reporting uterotonic use outside of CEmONC (OR = 3 · 0, 95 % CI = 2 · 4,3 · 7). This increased risk is particularly high for fresh stillbirths (OR = 4 · 0, 95 % CI = 3 · 0,5 · 3) and intrapartum-related causes of early neonatal deaths (birth asphyxia) (OR = 3 · 1, 95 % CI = 2 · 2,4 · 5).

**Conclusions:**

In this sample, outside uterotonic use was associated with substantially increased risk of fresh stillbirths, deaths due to birth asphyxia, and all perinatal deaths. In settings of high uterotonic use outside of controlled settings, substantial improvement in both stillbirth and early neonatal mortality may be made by reducing such use.

**Electronic supplementary material:**

The online version of this article (doi:10.1186/s12978-016-0241-x) contains supplementary material, which is available to authorized users.

## Plain English summary

Uterotonics can be used safely to treat disorders of labor in a delivery hospital where the mother can be monitored and complications can be treated. But in rural Bangladesh, uterotonics are also widely available and are often used outside of such controlled settings. We found that more babies were born dead or died during their first week of life if their mothers told us that they had received uterotonics before arriving at a safe delivery hospital. If fewer mothers received uterotonics to speed labour outside of controlled settings, many babies’ deaths can potentially be prevented.

## Background

Globally each year, an estimated 2 · 6 million newborns die [1], and another 2 · 6 million babies are stillborn, half of whom die during labour and birth [2, 3]. The vast majority (>98 %) of these deaths occur in low- and middle-income countries [3, 4]. With other causes of under-five mortality declining, neonatal deaths now account for 44 % of all under-five deaths making them a crucial focus for improving child survival [5–7]. Renewed calls to action to end these largely preventable child deaths include addressing gaps in knowledge about stillbirth risk factors and stillbirth prevention as well as preventing deaths due to birth asphyxia [3, 7, 8].

In recent years, practitioners in low- and middle-income countries have raised concerns that the use of uterotonics, an otherwise life-saving treatment, outside of controlled settings is contributing to these perinatal deaths [9]. Induction and active management of labor with uterotonics is a well-established and safe medical procedure as long as it is conducted in tightly controlled situations where one can identify correct indications of risk, precisely administer doses, frequently monitor fetal heart rate [9, 10] and perform emergency Cesarean section when necessary, namely in a CEmONC facility that follows appropriate procedures. In addition to facilitating active management of labor and reducing the complications of prolonged labour, uterotonics are also an important tool in preventing and treating post-partum haemorrhage, a leading cause of maternal death in low- and middle-income countries. Uterotonics can save lives and manage risk in clinical settings, but they can also threaten the lives of mothers and babies when used to augment or induce labor in settings without the appropriate resources and adherence to protocols. Such situations include home birth settings and basic EmONC facilities without full CEmONC support (henceforth, “outside uterotonic use”). These outside utertonics are frequently administered either as a bolus intravenous infusion without strict control of the infusion rate or as intramuscular injections. Notable consequences of “outside uterotonic use” include fetal hypoxia, neonatal encephalopathy or death, uterine rupture, and maternal death [9]. However, little systematic evidence has estimated the relative contribution of outside uterotonic use to perinatal outcomes [11], and currently none exists for stillbirth. The perceived benefit to the family of outside uterotonics use in Bangladesh includes speeding up labor, shortening labor duration, facilitating delivery, avoiding hospital delivery and reducing maternal suffering [12]. Given estimates of home-based uterotonic use ranging from 10 to 68 % in low- and middle-income settings [9–13], there have been a number of recent calls for rigorous studies to quantify the magnitude and effect of inappropriate augmentation on perinatal and maternal outcomes [9–12, 14, 15].

This cross-sectional study uses seven years of hospital records in a low-income setting in rural, northwestern Bangladesh to estimate the increased risk of several perinatal birth outcomes, including stillbirths, from the reported use of injectable uterotonics to induce or augment labor outside of CEmONC facilities. We test the hypothesis that outside uterotonic use will have negative effects on several, but not all birth outcomes. Specifically, it should increase the risk of intrapartum (i.e. “fresh”) stillbirth but not antepartum stillbirths (i.e. “macerated”). It should also increase the risk of early neonatal death (early NND) especially death caused by birth asphyxia. We assess this hypothesis against the null hypothesis of no increased risk among those mothers administered outside uterotonics.

## Methods

### Setting

LAMB Hospital is a CEmONC facility serving the rural poor in Northwest Bangladesh. On average 3500 babies are delivered at the hospital each year. During the study period, 5.7 % of all mothers experienced prolonged labor, 3.1 % breech deliveries, and 1.6 % eclampsia. Approximately one fifth (21.7 %) were treated with Cesarians. The median birthweight was 2.8 kg (1st quartile = 2.5 kg, 3rd quartile = 3.1 kg). The hospital has developed and trained medical providers in the use of a simple interactive checklist medical record system “circle sheets” for documenting key maternal and neonatal clinical information before, during and after delivery including history, examination, and diagnoses. In addition to facilitating real-time medical decision-making, the circle sheets are then entered into a customised database, checked and cleaned for use in standard reporting and clinical audits. For variables considered here, circle sheets with fixed responses require the practitioner to specify at least one response (e.g. Yes, No, or Unknown), and missing data are relatively rare. All data analyses here are generated by an ongoing hospital data system that was originally developed in 2006 along with a number of quality monitoring, feedback and training practices aimed at improving completeness and accuracy of data by midwives and clinicians. These include cross-comparing data entry, regular checks on completeness of data, on-ward observations of form completion, and regular retraining personnel It also involves frequent data reports to hospital staff and communication with staff about any changes in the understanding or completion of the interactive checklist records.

### Sample

**Fig. 1:**
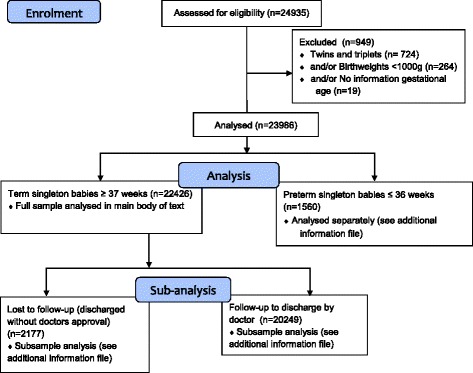
Sampling flow diagram

There were 24935 babies delivered at LAMB Hospital between January 1st, 2009 and December 31st, 2015. For these analyses, we exclude twins and triplets (2 · 9 % of births, *n* = 724) as well as babies with birthweights <1000 g (1 · 1 % of births, *n* = 264) and without information on gestational age (*n* = 19) (Total excluded *n* = 949). Pre-term births have a higher risk of adverse outcomes from prematurity related causes. Thus, we analyse pre-term births (≤36 week) separately (6 · 3 % of total births, *n* = 1560) and present these analyses in the additional information (see Additional file 1: Table S1). Pre-term births were classified as ≤36 week gestation using a validated method based on 8 external characteristics [16]. This leaves a final total sample of 22426 term singleton births ≥37 weeks and ≥1000 g. Of these 22426 births, 2177 (9 · 7 %) were admitted infants whose guardians discharged them without doctor’s approval, and thus were not available for follow-up in the case of complications. Analyses excluding these early discharged newborns (*n* = 20249) are not substantially different from those with the full sample (see Additional file 1: Table S2 for results on this subsample). We report results for the full sample of 22426 singleton term births in the main body. The flow diagram in Fig. 1 summarizes the excluded babies and the analysis pathways.

#### Variables

##### Birth outcomes

We examined five birth outcomes prior to discharge: (1) fresh stillbirth, (2) macerated stillbirth, (3) early neonatal death (before discharge), (4) early neonatal death due to intrapartum-related complications (birth asphyxia), as well as a (5) composite outcome of perinatal death (stillbirth and early neonatal death).

Stillbirth type (fresh or macerated) was determined by the midwife or doctor who delivered the baby and recorded in the mother’s chart and perinatal death form at the time of birth. Live born babies were either admitted to routine postnatal care by midwives or admitted under pediatric doctors according to strict clinical criteria including: sepsis risk, less than 2 kg birth weight, specific maternal risk factors, growth restriction, significant jaundice, congenital abnormalities, respiratory distress, weight loss >10 %, resuscitation needed after birth, and feeding difficulties among other indications. Infants that died immediately after birth were assessed and the cause of death documented. The 67 % of infants admitted to the hospital for routine postnatal care by midwives were determined free of these problems after a postnatal check. Each of the 33 % of infants admitted to the hospital under pediatric doctors was classified as either alive at discharge or early neonatal death (death within 7 days of delivery prior to discharge). Admitted babies length of stay was median 2 · 0 days and mean 2.5 days. For all early neonatal deaths, a senior obstetrician and senior pediatrician reviewed the mother and baby’s case notes as part of a monthly perinatal death audit. Causes of death were coded by these senior clinicians with the “Perinatal Problem Identification Programme (PPIP)”, originally designed and developed in South Africa as a facility audit tool for perinatal deaths [17]. The PPIP has 5 subcodes for hypoxia (hypoxia, hypoxic ischemic encephalopathy, meconium aspiration, persistent fetal circulation and hypoxia other) which we attribute to intrapartum-related complications (birth asphyxia). All data was extracted from the hospital database, Flow Information System Hospital or FISH [18].

##### Reported outside uterotonic use

At admission to the CEmONC unit, all mothers were routinely asked by the admitting midwife or doctor whether intramuscular injections or drips had already been administered to increase labor pains before arrival at this hospital. Mothers come to this CEmONC either from home or local basic EmONC facilities and only rarely are they given referral papers with details of treatment administered. Injectable uterotonics such as oxytocin are widely available in Bangladesh and use outside the CEmONC is usually by an intramuscular injection or in an intravenous infusion or drip without any monitoring [12]. If mothers stated they had received injections or drip prior to coming to the hospital, either affirmatively or in terms of the number of treatments, this was documented on their clinical records and subsequently entered into the database coded as 1 for “outside uterotonic use”. Otherwise they were coded as 0. It was not documented whether the outside injectable uterotonic use had been in the home or at a Basic EmONC facility. Nor was the provider of the outside uterotonic documented eg the Village Doctor - a type of unqualified allopathic provider who usually operates in the home setting [12] or a medically trained provider in the basic EmONC setting.

##### Covariates

We considered five potential covariates—prolonged labor, pre-eclampsia, eclampsia, induction or augmentation by uterotonics in the CEmONC hospital setting (LAMB Hospital), and Cesarian section.

Prolonged labor is a potential confounder of any observed relationship between outside uterotonic use and birth outcomes as prolonged labor can both: (1) lead caregivers to seek uterotonics in the home or in non-comprehensive EmONC facilities and (2) precipitate birth complications including intrapartum-related neonatal complications (birth asphyxia). For approximately 90 % of births, health workers documented information on when the mother’s first stage of labor had begun. This information was from mother’s self-report if she presented at the hospital already in the first stage of labor or from the partograph if her first stage started after admission. The duration of labor was calculated by subtracting the beginning of first stage labor from the time of delivery. A categorical prolonged labor variable was created with labor <12 h = 1, labor 12–24 h = 2, labor >24 h = 3, and labor time is missing = 4.

Eclampsia and pre-eclampsia are additional complications that may be confounding variables between outside uterotonic use and deaths due to birth asphyxia. Thus, we control for the effect of two levels of pre-eclampsia (moderate and severe) and eclampsia diagnosed by a clinical checklist. In this context, both CEmONC facility uterotonics to induce or augment and Cesarian section may arise from prolonged labor which could both precipitate outside uterotonic use prior to arrival at the EmONC facility and increase the risk of perinatal mortality. Thus we include these two procedures as proxies for potential confounders. Uterotonic induction or augmentation in LAMB Hospital is initiated by an Obstetric doctor for inadequate progress on the partograph. Uterotonic induction or augmentation at LAMB Hospital is documented in the maternal notes and entered on the database as a categorical variable: Yes, No or Unknown. Intermittent fetal heart monitoring is performed every 15 min for labors being induced or augmented with uterotonics. Cesarian section was recorded in the maternal birth chart and entered on the database as 1 = Cesarian section and 0 = no Cesarian section.

#### Analysis

We report raw rates of five outcomes—fresh stillbirth, macerated stillbirth, early neonatal death (before discharge), deaths due to birth asphyxia, and a fifth composite outcome of total perinatal deaths—for both the entire sample and the sub-sample reporting uterotonic use outside of CEmONC. We use a logistic regression to estimate the effect of outside uterotonic use on these outcomes, reporting both a crude, unadjusted odds ratios and odds ratios adjusted for potential covariates— prolonged labor, pre-eclampsia, eclampsia, uterotonic administration at the hospital, and Cesarian section. There were no cases of macerated stillbirth when a mother experienced eclampsia, so that variable was not included in the final macerated stillbirth model. Analyses were conducted in SPSS 22 · 0.

We estimated the population attributable risk percent from outside uterotonics using formula 4 in Rockhill and Newman applied to the adjusted relative risk [19]. Specifically, we calculate the population attributable risk percent $pd\left(\frac{RR-1}{RR}\right)$ where pd is the proportion of cases exposed to the risk factor and RR is the risk ratio associated with that exposure. The prevalence of all outcomes is less than 5 % in the population. Thus, we use adjusted odds ratios as approximations for adjusted relative risks in this calculation.

## Results

### Descriptives

Table 1 describes key outcomes and predictors for: (1) all singleton term births and (2) the sub-group of deliveries where outside uterotonic use was reported.

**Table 1:** Description of singleton term births

	All Singleton term births ≥37 weeks *n* = 22426	Group reporting outside uterotonics *n* = 1260
Perinatal Deaths		
Fresh Still Births	361 (1 · 6 %)	63 (5 · 0 %)
Macerated Still Births	191 (0 · 9 %)	8 (0 · 6 %)
Unspecified Still Births	5 (0 · 0 %)	3 (0 · 2 %)
Total early Neonatal deaths	318 (1 · 4 %)	53 (4 · 2 %)
Neonatal deaths from Birth Asphyxia	229 (1 · 0 %)	41 (3 · 3 %)
Total Perinatal deaths	875 (3 · 9 %)	124 (9 · 8 %)
Birth Complications		
Prolonged labor (>12 h)	1289 (5 · 7 %)	185 (14 · 4 %)
Pre-eclampsia (Moderate)	63 (0 · 3 %)	5 (0 · 4 %)
Pre-eclampsia (Severe)	303 (1 · 4 %)	12 (1 · 0 %)
Eclampsia	261 (1 · 2 %)	13 (1 · 0 %)
Hospital Procedures		
Cesarian-sections	4953 (21 · 1 %)	484 (38 · 4 %)
Cesarians due to fetal compromise	1501 (6 · 7 %)	97 (7 · 7 %)
Uterotonic to augment or induce	7516 (33 · 5 %)	435 (34 · 5 %)

% of births in parentheses

### Prevalence of outside uterotonic use

5 · 6 % of the women reported using an outside uterotonic, either as an injection or drip prior to arriving at the CEmONC facility. Prolonged labor greatly increased the reported use of uterotonics. For labors reported as lasting less than 12 h, 5 · 1 % of women reported using outside uterotonics (3 · 4 % injections and 4 · 4 % drip). For prolonged labors reported as lasting at least 12 h, these proportions rose substantially: 14 · 7 % reporting an outside uterotonic, 11 · 6 % reporting injections specifically, 13 · 0 % reporting a drip specifically.

### Association of outside uterotonic use with birth outcomes

The crude odds ratios for the association of uterotonic use outside CEmONC with 3 of the 4 birth outcomes were substantial and statistically significant—Fresh stillbirth OR = 3 · 8 95 % CI (2 · 8, 4 · 9), early NND OR = 2 · 9 (2 · 1,3 · 9), early NND due to Birth Asphyxia OR = 3 · 8 (2 · 7,5 · 3) (all *p* < 0.001). These are the birth outcomes that specifically relate to intrapartum events. The odds ratio for the remaining birth outcome, macerated stillbirth, which relates to the baby dying before the onset of labor was not found to be statistically associated with outside uterotonic use OR = 0 · 7 (0 · 4,1 · 5) (*p* > 0.10). The crude odds ratio for the composite birth outcome perinatal death was substantial and statistically significant OR = 3 · 0 (2 · 4,3 · 6) (*p* < 0.001) (Table 2).

**Table 2:** Crude odds ratios for four perinatal outcomes and total perinatal mortality

	Odds Ratio (95 % CI)
Fresh Stillbirth	3 · 7* (2 · 8,4 · 9)
Macerated Stillbirth	0 · 7 (0 · 4,1 · 5)
Early NND	2 · 9* (2 · 1,3 · 9)
Early NND due to Birth Asphyxia	3 · 8* (2 · 7,5 · 3)
All Perinatal Death (Composite)	3 · 0* (2 · 4,3 · 6)

Notes: *p < 0.001

Table 3 presents the model-adjusted odds ratios. After controlling for all covariates, there is an increased risk of all categories of perinatal mortality if the mother had a diagnosis of either prolonged labor, moderate or severe pre-eclampsia, and eclampsia. There was no significant associated between LAMB Hospital administered uterotonics and any perinatal outcomes. There was a decreased risk of fresh stillbirth and an increased risk of early neonatal death and death due to birth asphyxia with Cesarian sections. Overall, Cesarian sections were associated with lower risk of perinatal mortality (OR = 0.7 (0.5,0.8) (*p* < 0.001).

**Table 3:** Logistic Regressions predicting five birth outcomes from reported outside uterotonics, adjusted odds ratios (singleton, term births ≥ 37 weeks, *n* = 22426)

	Outcome				
Predictor	Fresh Stillbirth	Macerated Stillbirth	Early Neonatal Death	Intrapartum causes (Birth Asphyxia)	All Perinatal Deaths
Outside uterotonics	4 · 0*** (3 · 0,5 · 3)	0 · 8 (0 · 4,1 · 7)	2 · 9*** (2 · 1,4 · 0)	3 · 1** (2 · 2,4 · 5)	3 · 0*** (2 · 4,3 · 7)
Labor duration					
<12 h	ref.	ref.	ref.	ref.	ref.
12–24 h	3 · 1*** (2 · 1,4 · 4)	1 · 9* (1 · 0,3 · 5)	1 · 9** (1 · 3,2 · 9)	2 · 1** (1 · 3,3 · 3)	2 · 5*** (1.9,3.2)
>24 h	2 · 5*** (1 · 5,4 · 3)	1 · 7 (0 · 7,3 · 8)	1 · 0 (0 · 4,2 · 3)	1 · 1 (0 · 5,2 · 8)	1 · 8** (1 · 2,2 · 6)
Missing	2 · 4*** (1 · 5,3 · 9)	2 · 6* (1 · 1,6 · 0)	1 · 3 (0 · 9,1 · 9)	1 · 2 (0 · 8,1 · 9)	1 · 7*** (1 · 3,2 · 3)
Pre-eclampsia					
none	ref.	ref.	ref.	ref.	ref.
moderate	2 · 0 (0.5,8.4)	1 · 7 (0.2,12.2)	2 · 0 (0.5,8.2)	2 · 8 (0.7,11.5)	2 · 0 (0.8,5.0)
severe	4 · 5*** (2.7,7.6)	3 · 0** (1.5,6.3)	2 · 6** (1.4,4.7)	3 · 3*** (1.8,6.2)	3 · 6*** (2.5,5.1)
Eclampsia	4 · 7*** (2.8,7.9)	NA	4 · 7*** (2.8,7.8)	5 · 6*** (3.2,9.7)	3 · 7** (2.5,5.4)
Hospital-based Uterotonic Augmentation					
No	ref.	ref.	ref.	ref.	ref.
Yes	1 · 0 (0 · 5,1 · 9)	2 · 4 (0 · 5,10 · 9)	0 · 6 (0 · 4,1 · 1)	0 · 7 (0 · 3,1 · 4)	0 · 8 (0 · 5,1 · 3)
Unknown	1 · 2 (0 · 6,2 · 5)	5 · 8* (1 · 3,27 · 0)	0 · 8 (0 · 5,1 · 5)	1 · 1 (0 · 5,2 · 3)	1 · 3 (0 · 8,2 · 0)
Cesarian Section					
No	ref.	ref.	ref.	ref.	ref.
Yes	0 · 3*** (0 · 2,0 · 4)	0 · 1*** (0 · 0,0 · 2)	2 · 0*** (1 · 6,2 · 6)	2 · 1*** (1 · 6,2 · 8)	0 · 7*** (0 · 5,0 · 8)

Adjusted Odds Ratio with 95 % CI in parentheses, adjusted for length of labor, hospital-based uterotonic augmentation or induction, pre-eclampsia, eclampsia, and Cesarian section

Note: *ref* reference category

***p < 0.001, **p < 0.01, *p < 0.05

When adjusting for prolonged labor, pre-eclampsia, eclampsia, hospital administration of uterotonics, and Cesarian section, the associations of all perinatal mortality outcomes with outside uterotonic use remained significant and substantial, with the exception of macerated stillbirths (Table 3).

### Estimating population attributable risk

If these statistical associations reflect the causal impact of outside uterotonic use on perinatal mortality with no unmeasured confounding or omitted variables, then the estimated percentage of deaths due to exposure to uterotonics outside the CEmONC setting is 10 % of early neonatal deaths, 11 % of birth asphyxia deaths, 10 % of perinatal deaths and 14 % of fresh stillbirths. These estimates are based on the current population with a relatively low rate of reported outside uterotonic use (5.6 %) compared to other published studies. If we adjust these estimates for higher rates of outside uterotonic use in other studies (10–68 % of home births) [9–13], then such uterotonic use would be associated with much greater percentage of all perinatal mortality—for example 28 % of perinatal deaths at 20 % births with outside uterotonic use or 36 % of perinatal deaths at 30 % outside uterotonic use.

## Discussion

In a CEmONC setting in Northwestern Bangladesh, we find that reported uterotonic use prior to arrival is associated with a substantially increased risk of perinatal mortality, including fresh stillbirth, early neonatal death prior to discharge, and death from birth asphyxia. This effect is independent of the potential confounding effect of prolonged labor, pre-eclampsia, eclampsia, CEmONC-administration of uterotonics and Cesarian section. Consistent with the expectation that outside uterotonic use increases the risk for perinatal mortality, there is no association with macerated stillbirth which would in most cases result from antepartum fetal death that precedes uterotonic use in labour. The findings about neonatal outcomes confirm results of a recent study in another South Asian setting showing similar relationships between unmonitored uterotonic use and neonatal mortality and morbidity [11]. However, by estimating the increased risk of fresh stillbirth associated with outside uterotonic use, our findings also highlight the potential impact of uterotonic use outside of CEmONC on all babies’ lives during the entire continuum of intrapartum and postnatal life.

In this setting, we estimate that outside uterotonic use accounts for 10 % of all perinatal deaths, including 11 % of birth asphyxia deaths and 14 % of fresh still births. However, in both hospital- and community-based surveys conducted in the hospital catchment area, this population has substantially lower rates of reported outside uterotonic use than other populations in South Asia and low-income countries (5 · 6 % and 2.3 to 23.5 % in representative surveys of recent births in the hospital source communities compared to 10–68 % in other studies) [9–13]. If we assume outside uterotonic use is associated with the same risk of perinatal mortality as in a setting with higher rates of home-based births use uterotonics, then we estimate much higher proportions of perinatal deaths would be due to uterotonics outside a facility with appropriate controls in place (e.g., 28 % of perinatal deaths with 20 % use).

Although this study showed no evidence of increased risk from uterotonics administered after arrival at this CEmONC facility, it is important to point out that delivery in a facility designated as CEmONC may not be synonomous with appropriate control. Specifically, it is crucial to have controls in place for appropriate monitoring and regulation of the uterotonic infusion including, monitoring uterine contractions, fetal heart-rate, use of the partograph as a decision making tool and immediate access to Cesarian section when needed . Not all CEmONC facilities may adhere to these standards in practice. These findings also show that even in a CEmONC facility with the capacity to provide a Cesarian-section within 20 min of admission, as is the case in this study, there are still a remarkable number of perinatal deaths which may be due to the sequelae of prior outside uterotonic use.

These findings must be interpreted in light of a number of data limitations. Reporting in locally meaningful language of “injections and drips to increase labor pains” is only a proxy for actual use of uterotonics outside the CEmONC facility. It is likely subject to underreporting to hospital staff due to either self-presentation bias on the part of patients or failure to ask and / or document on the part of staff. It is possible that some of the drips administered outside were plain intravenous fluids and did not contain uterotonics. Nonetheless, the proxy measure for uterotonic use is strongly associated in the expected direction with perinatal outcomes, suggesting that it is a suitably valid measure of outside uterotonic use. Assessing the stage in labour that the mother presented to the CEmONC hospital and at which stage the outside jnjectable uterotonics were used as well as the dose or frequency used were beyond the scope of this study.

The deliveries at LAMB hospital also represent a selective sample of all those deliveries in the surrounding area [20, 21]. They likely include more complications and fewer normal deliveries than occur in the surrounding population, and thus leave open questions about the generalizability of the specific estimates or qualitative findings to the broader population. Recent efforts to monitor outside uterotonic use and perinatal mortality for all deliveries in the hospital’s surrounding area will hopefully shed new light on the generalizability of these findings.

It is also possible that prolonged labor still confounds the relationship between outside uterotonic administration and perinatal mortality, but that error in assessing prolonged labor leads to incomplete detection of statistical confounding. However, the fact that adding an assessment of prolonged labor does almost nothing to change the estimated effect of outside uterotonic use on perinatal mortality partially alleviates this concern.

## Conclusions

We find that reported use of uterotonics prior to arriving at the comprehensive facility is associated with a substantially increased risk of perinatal mortality. These findings point to the potentially substantial improvement in perinatal mortality to be made by reducing the currently widespread use of uterotonics for induction and augmentation outside of CEmONC facilities in low- and middle-income countries. Such efforts would benefit from systematic surveillance of such use as well as studies that identify and target the specific pathways by which uterotonics come to be administered outside of CEmONC facilities. These will crucially involve a better understanding of the way that local availability, perceived medical need, and family demands for a quick delivery play a role in guiding decisions to use uterotonics outside of CEmONC facilities.

## Additional file

Additional file 1: Perinatal mortality associated with use of uterotonics outside of Comprehensive Emergency Obstetric and Neonatal Care: a cross-sectional study. (DOCX 34 kb)

## Abbreviations

CEmONC: Comprehensive Emergency Obstetric Neonatal Care
early NND: early Neonatal Death (day 0–7)
EmONC: Emergency Obstetric and Neonatal Care
FISH: Flow Information System Hospital database
MIS-Research: Management Information Systems – Research Department
PPIP: Perinatal Problem Identification Programme
