# Supplementary material for: Perinatal mortality associated with use of uterotonics outside of Comprehensive Emergency Obstetric and Neonatal Care: a cross-sectional study
Source: Reprod Health. 2016 Oct 6;13:129. doi: 10.1186/s12978-016-0241-x (PMC5054615; doi:10.1186/s12978-016-0241-x)
Supplement: Additional file 1: — Perinatal mortality associated with use of uterotonics outside of Comprehensive Emergency Obstetric and Neonatal Care: a cross-sectional study. (DOCX 34 kb) [file 12978_2016_241_MOESM1_ESM.docx]

Additional file 1: Perinatal mortality associated with use of uterotonics outside of Comprehensive Emergency Obstetric and Neonatal Care: a cross-sectional study

Louise T Day MRCPCH,^1,2^ Daniel Hruschka PhD,^3^ Felicity Mussell MRCOG,^4^ Eva Jeffers,^3^ Stacy L Saha MSc,^1^ Shafiul Alam MA^1^

^1^LAMB MIS-Research Department, Parpatipur, Dinajpur 5250 Bangladesh, ^2^LAMB Hospital Pediatric Department, Parbatipur, Dinajpur 5250 Bangladesh, ^3^Arizona State University, Tempe, AZ 85287, USA ^4^LAMB Hospital Obstetric Department, Parpatipur, Dinajpur 5250 Bangladesh

**Descriptives**
Table S1 describes key outcomes and predictors for all births, singleton term births and singleton pre-term births. Our analyses of pre-term births exclude babies with birth weight less than 1000g (0·9% of all births, n = 96) according to one criterion for reporting perinatal mortality in the international setting [1].

**Table S1. Full Description of births.** % of births in parentheses.

|  |  | Singleton Births ≥ 1000g | |
| --- | --- | --- | --- |
|  | All births  (n = 24935) | Term births ≥ 37 weeks  (n = 22426) | Pre-term births  (n = 1560) |
| **Outside Uterotonics** | 1386 (5·6%) | 1260 (5·6%) | 80 (5·1%) |
| **Birth details** |  |  |  |
| 28-36 w or < 1000g^a^ | 2152 (8·6%) | na | na |
| Twin & triplet births | 724 (2·9%) | na | na |
| **Perinatal Mortality** |  |  |  |
| Fresh Stillbirths | 578 (2·3%) | 361 (1·6%) | 139 (8·9%)** |
| Macerated Stillbirths | 403 (1·6%) | 191 (0·9%) | 126 (8·1%)** |
| Unspecified Stillbirths | 10 (0·0%) | 5 (0·0%) | 4 (0·3%) |
| Early Neonatal deaths | 625 (2·5%) | 318 (1·4%) | 157 (10·1%)** |
| Birth Asphyxia deaths | 270 (1·1%) | 229 (1·0%) | 37 (2·4%)** |
| Total Perinatal deaths | 1616 (6·5%) | 875 (3·9%) | 426 (27·3%)** |
| **Birth Complications** |  |  |  |
| Prolonged labor (> 12 h) | 1413 (5·7%) | 1289 (5·7%) | 78 (5·0%)* |
| Pre-eclampsia (Moderate) | 88 (0·4%) | 63 (0·3%) | 19 (1·2%)* |
| Pre-eclampsia (Severe) | 491 (2·0%) | 303 (1·4%) | 127 (8·1%)* |
| Eclampsia | 403 (1·6%) | 261 (1·2%) | 102 (6·5%)* |
| **Facility Treatments** |  |  |  |
| Caesarean -sections | 5407 (21·7%) | 4953 (21·1%) | 253 (16·2%)** |
| C-sections (fetal compromise) | 1608 (6·4%) | 1501 (6·7%) | 66 (4·2%)** |
| Uterotonic induction or augmentation | 8281 (33·2%) | 7516 (33·5%) | 479 (30·7%) |

^a^Includes 19 with missing gestational age.

Fisher's exact test of difference in proportions between singleton term births and singleton pre-term ≥ 1000g. * p < 0·05, ** p < 0·001.

**Results**

**Prevalence of outside uterotonics among singleton pre-term births** ≥ 1000g**.**

Among pre-term births ≥ 1000g, prolonged labor appears to be associated with an increase in outside uterotonic use, most notably via drip. For labors lasting less than 12 hours, 2·6% of women reported injections to increase labor pains, 4·7% reported intravenous saline, and 5·0% reported at least one of these prior to arriving at a comprehensive EmONC Facility. For labors lasting at least 12 hours, 1·3% reported injections, 7·7% reported intravenous saline, and 9·0% reported at least one of these in the home setting. However, values for prolonged labor among pre-term births should be interpreted with caution due to the small number of such births (n = 78).

**Association of outside uterotonics with birth outcomes among singleton pre-term births** ≥ 1000g**.**

Among pre-term births ≥ 1000g, the crude association of outside uterotonic use was not statistically significant for any of the single birth outcomes—Fresh stillbirth OR = 1·1 95% CI (0·6,2·3), Macerate stillbirth OR = 0·4 95% CI (0·1,1·4), Early NND OR = 1·1 (0·6, 2·3), Early NND due to Birth Asphyxia OR = 1.7 (0.5, 5.5), composite measure of perinatal death OR = 0·8 (0·5,1·3). These results indicate that the large numbers of deaths among pre-term births are likely due to causes other than outside uterotonic use.

**Table S2. Crude (unadjusted) and adjusted Odds Ratios (OR) predicting five birth outcomes from outside uterotonic use (singleton, term births ≥ 37 weeks excluding babies without official discharge, n=20249).**

|  | Unadjusted  OR (95% CI) | Adjusted  OR (95% CI) |
| --- | --- | --- |
| **Fresh Stillbirth** | 3·8* (2·9,5·1) | 4·4* (3·3,5·8) |
| **Macerated Stillbirth** | 0·8 (0·4,1·5) | 0·9 (0·4,1·8) |
| **Early NND** | 3·5* (2·6,4·7) | 2·9* (2·1,4·0) |
| **Early NND due to Birth Asphyxia** | 3·9* (2·8,5·7) | 3·3* (2·3,4·7) |
| **All Perinatal Death (Composite)** | 3·1* (2·5,3·7) | 3·2* (2·6,3·9) |

Notes: *p < 0.001

Adjusted Odds Ratio adjusted for length of labor, pre-eclampsia, eclampsia, hospital-based uterotonic induction or augmentation, and Caesarean section

**References**

[1] Richardus JH, Graafmans WC, Verloove-Venhorick SP, Mackenbach JP (1998) The Perinatal Mortality Rate as an Indicator of Quality of Care in International Comparisons. Medical Care; 36: 54-66.
